# Report of the Indo-US Health Care Summit 2009 - Mental Health Section

**DOI:** 10.4103/0019-5545.58298

**Published:** 2009

**Authors:** Anand K. Pandurangi, Nimesh G. Desai

**Affiliations:** Virginia Commonwealth University, Richmond, Virginia, USA; 1 Department of Psychiatry, Institute of Human Behavior and Allied Sciences (IHBAS), Delhi, India

**Keywords:** Indo-US collaboration, medical education, medicine psychiatry integration, public education

## Abstract

The 2nd Indo-US Health Care Summit held in January 2009 was a forum to discuss collaboration between physicians in the US and India on medical education, health care services and research. Six specialties were represented including Mental Health (MH). Using Depression as the paradigmatic disorder, the following objectives were developed.

**Objective I – Leadership and Public Education:**

Linkage with like-minded agencies and organizations. The core message should be simple. Major Depression is a brain disorder. Depression is treatable. Timely treatment prevents disability and suicide.

**Objective II – Medical Education:**

To improve psychiatric education, it was proposed that (1) relations between US/UK and Indian mid-level institutions be established, (2) teaching methods such as tele-psychiatry and online courses be pursued, (3) use models of teaching excellence to arouse student interest, and (4) develop core curricula for other branches of medicine, and CME.

**Objective III - Reduce Complications of Depression (Suicide, Alcoholism):**

Goals include (1) decriminalizing attempted suicide, (2) improving reporting systems, and including depression, psychosis, alcoholism, and suicide in the national registry, (3) pilot studies in vulnerable groups on risk and interventions, and (4) education of colleagues on alcoholism as a link between psychiatric and medical disorders.

**Objective IV - Integrating MH Treatment & Primary Health Care:**

The focus should be on training of general practitioners in psychiatry. Available training modules including long distance learning modules to be suitably modified for India.

Collaborations and specific project designs are to be developed, implemented and monitored by each specialty group and reviewed in future summits.

## INTRODUCTION

The Indo-US Health Care Summits are a forum to discuss collaborative initiatives between physicians in the US and India to address medical education, health care services and scholarly initiatives. The second Annual Indo-US Health Care Summit (HCS) was held in New Delhi, India from January 2-4, 2009. Six disease specialties were the focus of this summit - Allergy and Immunology, Cardiology, Diabetes, Infectious Disease, Maternal and Child Health, and Mental Health. This report pertains to the mental health (MH) section. The report is a summary of the reviews, deliberations and conclusions of the mental health panel.

The summits are sponsored by the American Association of Physicians from India (AAPI) and the Indian Medical Association (IMA), in consultation with the Government of India, Ministry of Health and Family Welfare (GOI) and the Medical Council of India (MCI). The first summit was held in New Delhi in December 2007. Other overseas organizations of physicians of Indian origin, such as those from the UK, Canada and Australia have evidenced interest in the summit process and were invited as observers for the second summit. Each specialty organized a panel of delegates from the US and India. Nominations were submitted to the sponsoring organizations and the section chairs. Delegates were selected through an informal process based on interest, expertise and availability. The MH panel membership is included at the end of this report.

## BACKGROUND

The MH panel of the first HCS had identified Depression as the paradigmatic disorder to understand the challenges and opportunities available in mental health.[1] Depression ranks amongst the top 10 causes of disability, worldwide.[2] Suicide is a complication of depression. Suicide rates in India have been reported in the range of 98–118 per 100,000. These are likely underestimates.[3–6] Depression affects or complicates all major medical disorders, especially cardiovascular morbidity and mortality.[7] Effective treatments are available for this disorder with nearly 80% response and >60% remission rates with sequential treatment.[8,9] The following were identified as potential areas for collaboration between Indian and Indo-American Psychiatrists.

- Depression Education – Leadership and the Public
- Medical Education- Undergraduate, Postgraduate and Continuing Medical
- Integration of Psychiatric Care with Primary Medical Care
- Reducing/Preventing Complications of Depression such as Suicide and Alcoholism
- Reducing Stigma and Increasing Access

### The second HCS

The primary task of this summit was to develop short- and long-term recommendations in pursuit of the above objectives. Many goals emerged from the deliberations during the summit and are outlined below. However, it was articulated during the summit, that many of the goals were being mentioned only to highlight their importance and for the sake of comprehensiveness. The summit delegates, and any work-groups that might emerge from this and future summits, could not possibly achieve all the goals that were articulated, working in isolation. Achievement of the broader agenda would need a mammoth and unprecedented effort in mental health in India. Further, it was fully recognized that multiple efforts are underway within India on each of the topics identified by the panel. Specifically, it was agreed that the summit leadership needed to work with the Indian Psychiatric Society (IPS), leading academic departments of psychiatry in India, South Asia section of the World Health Organization (WHO), non-governmental agencies (NGO) and non-profit MH organizations, and the corporate health sector in India, as well as interested overseas organizations to harness a synergy in our efforts. It was noted that considerable experience exists in other countries outside of US and India on the issues and goals of the second summit, and successful models from these countries could inform the development of India-specific models. The interest shown by physician organizations such as the Indo-American Psychiatric Association (IAPA) and the British Indian Psychiatrist Association (BIPA) in becoming part of the coalition to achieve the summit goals was particularly appreciated. Since the summit, there are efforts underway for the establishment of a global coalition oriented towards Indian Psychiatry and such an organization could serve as an umbrella entity for the pursuit of the objectives.

The objective of reducing stigma listed in the outcome of the first HCS was subsumed under the broader objective of public and leadership education, and the objective of increasing access was subsumed under the objectives of training and education, and integrating psychiatric care with primary medical care. For each of the remaining topics, a lead presenter from the US and India led the discussion. The background material on each objective was reviewed and following focused discussion, broad conclusions were reached. From these, specific goals were developed. In preparation of this report, additional reviews were conducted by the lead-author, and have been incorporated into the review for each objective.

Before tackling the specific objectives, the panel also felt it is important to emphasize some generic messages about psychiatry, psychiatrists and psychiatric disorders, such as:

- Psychiatrists are specialists in psychiatric medicine. Psychiatric therapies, especially pharmacotherapy and behavior therapy are evidence-based, making psychiatry a scientific and well-established branch of medical sciences.
- Stigma around mental health issues and psychiatric disorders is ubiquitous and a global issue of concern, and not limited to India.
- Not all psychiatric disorders are chronic. Many psychiatric disorders are short-term, episodic and self-limiting. Most psychiatric and behavior disorders respond well to treatment.
- Major depressive disorder is a brain disorder. Depression is not the fault of any individual. Depression is highly treatable with medications and psychotherapy. Suicide is often a complication of depression and can be prevented.

### Objective I - Public and leadership education

It was recognized that education of the public and leadership is a critical component and fundamentally needed to effectively address the challenges of Depression. Key leadership groups were identified as legislators, corporate leaders, community activists, educators, students, judiciary, law enforcement personnel, public and private administrators, philanthropists, religious and spiritual leaders, women's groups, and traditional and alternative health care providers. Even then, the panel felt this is not an exhaustive list. Finally, we can never lose sight of the fact that about 72% of India is rural (about 835 million).[10] Each primary health center (PHC) serves approximately 30,000 people and the medical officer is the de-facto leader from the health perspective. Medical officers in the PHC, Community Health Center (CHC) and District Health Center (DHC) network are critical targets in any effort to get the mental health messages across[11] (see more discussion on the strengths and weaknesses of this structure, under Objective IV).

It was suggested that the impetus and leadership for this effort should come from organized medicine (IMA, AAPI) and organized psychiatry (IPS, IAPA, etc). These organizations can help with the development and customizing of tools and techniques that MH advocates need to achieve this educational objective. We should build upon existing initiatives and programs including those developed and tested under the auspices of such organizations as the World Health Organization (WHO) and the World Psychiatric Association (WPA) rather than re-invent the wheel and duplicate efforts. For example, on the topic of public and community education including tackling the challenges of stigma, there are many successful projects that could be reviewed and customized to India. Some examples include: Mind Out for Mental Health, England, “See Me”, Scotland, “MindMatters”, Australia, “Stigma Watch: Media Education Campaign”, Australia, “Like Minds Like Mine”, New Zealand, “Open the Doors”, and the World Psychiatric Association (WPA) programs including in India and 19 other countries (www.openthedoors.com).[12–16] In India, the NGOs are playing a prominent role and many successful programs have been implemented over the last 20 years. These have been well-catalogued in a book by Patel and Thara.[17] Persons working in these organizations bring a deep sense of commitment and often speak from their heart. They tend to be person-centered, recovery-oriented and their views resonate well within the community. On the other hand, they often rely heavily on the efforts of a few charismatic and dedicated individuals, and may or may not sustain over a long period of time. Further, their resources tend to be limited, and often there is also limited to little coordination with government sponsored public health efforts.

Collaborations should be created with the academic departments, government agencies and the corporate sector to fund and implement demonstration projects and test what works and what does not within the Indian context, and more importantly what customization is needed to make projects successful elsewhere to work in the Indian context. For this, partnerships need to be established with user/care-giver and self-help groups, advocacy agencies, media, NGOs, the Rotary club, Lions club, cultural, religious and volunteer organizations.

It was suggested that in addition to proactive, grass roots education, situation and event responsive styles and strategies be utilized, particularly with legislators, government leaders, media-personalities and the for-profit corporate sector. As unfortunate as it was, the Erawadi incident of the inhumane treatment of persons with mental retardation also offered an opportunity for professionals to educate the public and the leadership on the plight of the mentally ill.[18] Likewise the untimely death of the Hindi film actress Parveen Babi raised the awareness of the press and the public regarding schizophrenia, suicide and the burden of mental illness.[19] It was noted that stigma raises its ugly head at particularly important stages of life, especially employment and marriage.[20,21] Interestingly, no stigma (or less stigma) appears to be attached to going to traditional places of healing. Modern psychiatry needs to understand the dynamics of help-seeking behaviors from this observation. Stigma is a complex phenomenon and simple assumptions about it don't hold up when critically examined.[22–24] It was observed that stigma is more of a barrier in the middle and upper classes and less so in the lower socioeconomic class.

MH leaders should seize the opportunity during major events in their area and participate to deliver the message. Classroom teaching is felt not to be particularly beneficial. IMA and IPS office bearers should be asked and encouraged to take a lead role in these efforts. It was noted that unfortunately, mental health support groups themselves are vulnerable to stigmatization. In several states in the US, including Florida, South Carolina and Oklahoma, a model that has worked well is “Partners in Crisis”. In this model, various groups such as MH providers, police, MH advocates and media come together for a quarterly meeting and review as well as plan.[25] As health insurance as a mode of paying for health care evolves in India, every effort should be made to include MH as a benefit. Funding under the National Mental Health Program (NMHP) of the GOI is in place[26] and the MH coalition should make every effort in ensuring the objectives of the summit are given their due share, as this program evolves and is implemented. For example, the goals of the revised NMHP include training personnel at PHC, CHC and DHC in mental disorder identification and treatment.[27] For this, vigorously tested models of such education, qualified trainers, training delivery systems, knowledge of the unique profiles of the end-user and the region in which this is to be implemented, etc are needed. Very little of these exist at this time. The so-called Bellary model of community care is based on a well-researched model.[28] Under the NMHP, it has been (partially) implemented in only about 110 districts, and its emphasis on serious mental illnesses is often perceived as excluding the common mental disorders (CMD) such as depression and anxiety disorders. Under the revised NMHP, 325 districts are expected to be covered,[27,29] and the proposed consortium will need to advocate for including its objectives, in such implementation. The programmatic elements of NMHP have also not kept up with the various developments in mental health, especially in the area of public education, public-NGO partnerships, manpower development, coverage of urban and semi-urban areas, especially integration with general medical practitioners, etc.[30,31] Likewise the efforts at education and de-stigmatization from the GOI are miniscule, such as few radio and TV spots in different languages.

In terms of timelines, in the early rounds, the target groups to focus on were identified as medical colleagues, police and judiciary, community leaders, legislators, medical officers and health workers including communicable disease surveillance (CDS) workers, and school and university teachers. Removing stigma within medical fraternity by collaborating with IMA in organizing events to showcase psychiatry as science and evidence-based medicine, was felt to be a necessary first step for this rather broad objective, and integrating mental health education with current health programs running in various media was emphasized as an early step to take. It is especially important that professionals make themselves available to the media to ensure accurate portrayal of mental illness and its impact on the individual, family and community.

Examples of resources, tools, techniques and activities to include but are not limited to:

1. Publication and distribution of booklets on MH and depression in different languages[32]
2. Hotlines to provide information on disease state, access to treatments, brief education on complications, methods of prevention, and a message of hope[33]
3. Call-in radio programs using successful models, for example on Radio Mirchi (India)[34]
4. MH camps[35]
5. Celebrities to tell their own stories on depression and other disorders, as has been successfully done in the US[36]
6. Movies with mental health themes and positive portrayal of mental health and psychiatry
7. Newspaper articles by professionals
8. Walkathons in support of mental illness[37]
9. Mental illness awareness week (MIAW)[38,39]
10. Legislative days to educate and lobby legislators
11. Involving other medical specialties to help in the missions of increasing awareness, early detection, accurate diagnosis and initiating treatment.

### Objective I - Goals

Create a workgroup with representatives from AAPI and IAPA (USA) and IMA and IPS (India). This group to work towards the development of a broad-based umbrella organization or consortium to include members of the four organizations, representatives of academic departments, other overseas organizations of physicians of Indian origin and likeminded NGOs, and various stakeholders in India as well as link-up with WHO and WPA, to develop a roadmap of education activities for each target group, starting with the early target groups noted above. Independent of the summit, an active effort has occurred for the creation of a global Indian psychiatry organization through the efforts of dedicated individuals and such an organization could become a nodal entity for the pursuit of this objective. Examples of resources, tools, techniques and activities to be considered by this consortium to include but are not limited to:

1. Publication and mass distribution of booklets on mental health, especially depression and anxiety disorders, in different Indian languages. This effort should co-opt the help of professional authors who have written extensively in local languages, and media writers and artists to achieve the widest reach possible.
2. Creation of hotlines to provide information on disease state, access to treatments, collect statistics on complications, impart methods of prevention, and deliver a message of hope.[26]
3. Creation and support of more call-in programs on broadcast media, using successful models.[34]
4. Use of celebrities to tell their own stories on depression and other disorders, as has been successfully done in the US and other countries.[36,40]
5. Distribution of recent feature films with mental health themes and positive portrayal of mental health and psychiatry, for example the movies “Devrai”, a Marathi language film, “Aagaatha” in the Kannada language, “15 Park Avenue” in Hindi, etc.
6. Increasing the number of articles by professionals in newspapers.
7. Encouragement and support of “mental health walks.”[37]
8. Conducting MH camps.[35]
9. Encouragement and support of a mental illness awareness week (MIAW).[38–40] It was noted in parts of the country that October 10^th^, which is identified as world mental health day has gradually emerged as the key day for mental health awareness activities, and this needs to be nurtured and supported vigorously. The core message through these efforts should be simple as enunciated in the first summit, and repetitive.
  - Major depression is a brain disorder.
  - Depression is a no-fault condition.
  - Depression is highly treatable.
  - Help is available.
  - Timely treatment will improve symptoms, enhance quality of life and prevent disability and suicide.

### Objective II – Undergraduate medical education

In reviewing this topic, reference was made to the deliberations of the first summit,[1] which had concluded that undergraduate (UG) medical education in psychiatry in many (but not all) medical colleges of India left much to be desired. The current summit focused more extensively on UG medical education in psychiatry. Preparatory reviews prior to the summit had included brief reviews of recommendations by earlier medical education committees, including the broad recommendations of the Bhore committee in 1946,[41] the Shrivastava committee in 1975,[42] the Bajaj committee in 1986,[43] the report by Kacker and Adkoli in 1993[44] and the report by Majumdar *et al*. in 2004,[45] as well as the proceedings of a comprehensive symposium on this topic at ANCIPS 2008 in Kolkata.[46] The latest MCI guidelines[47] on the required and recommended curricula were reviewed. The GOI has accepted psychiatry as a separate subject in UG training and this is awaiting MCI action. The MH panel asks that IPS robustly pursue this and get it implemented. Reference was made to the WPA-recommended core curriculum in UG psychiatry.[48] Papers from the ANCIPS symposium also reviewed UG education worldwide,[49] and referred to previous reviews and recommendations on UG psychiatry education from the seminar at the Central Institute in Ranchi in 1965, and workshops at JIPMER, Pondicherry in 1983, and AIIMS in 1994.[50] The urgent need for action was emphasized.[51] The article by Trivedi and Dhyani made specific recommendations to alleviate the present shortcomings,[52] and the article by Dale *et al*.[53] noted the importance given to psychiatric education in the UK with a few medical colleges offering 12 weeks of clinical psychiatry training, while also pointing out that much variability exists within the 27 medical colleges in UK.[53] The above body of work has thoroughly reviewed the current weaknesses in UG psychiatry training in India and elsewhere, and offered a curriculum and several solutions to implement the same. Broadly, the second HCS MH panel endorsed these recommendations.

MCI guidelines[47] require 20 hours of didactic teaching in psychiatry and related sciences, two weeks of clinical training of a minimum of 3 hours per day, and in the internship year, two weeks of clinical training under the Medicine rotations track, and two weeks of psychiatry elective rotation. In comparison, in the US, medical schools typically provide greater than 60 hours of didactics and other forms of learning including many active learning methods such as team-based learning, as well as six to eight weeks of clinical clerkships, with a broad variety of clinical exposures to inpatient, outpatient, consultation liaison, substance abuse, emergency psychiatry etc. Whereas it is neither desirable nor indicated to exactly replicate the American or UK training curricula, the duration of training, breadth and depth of training should be roughly equivalent, as the prevalence, nature and burden of mental disorders in India demands this.

Medical colleges in India may be described as trimodally distributed. Of the 262 medical colleges as of January 2009, the top 20 colleges appear to have sufficient psychiatry faculty and clinical facilities to provide a quality education. The second tier colleges numbering between 30-50 have one to three fulltime faculty and several adjunct faculty. The bottom 200 schools rely heavily on part-time clinical faculty and limited or borrowed clinical facilities. It was suggested that IMA and AAPI leaders endorse the current actions of IPS and MH advocates and advocate for facilities, lecturers, and examinations in psychiatry during UG training. However, given the extremely limited number of psychiatrists in the country even for patient care services, and the lucrative nature of private practice of psychiatry, it is unlikely that such endorsements and support alone will result in medical colleges having a full complement of faculty, in the foreseeable future. It is therefore imperative that alternative, realistic and creative solutions be developed. The summit panelists suggested a number of such initiatives. The suggested list below (a through k) is by no means comprehensive and serves more as a starting point for further deliberations and initiatives. The primary objective is to provide a solid education in psychiatry to all medical students, no matter which specialization they will pursue. Another objective is to accurately portray the state of the art in psychiatry and help develop an interest and enthusiasm in psychiatry among the new generation of doctors. This in turn is likely to have a positive impact on the earlier objective of public and leadership education. Again, in the pursuit of this objective as well, medical educators, academic psychiatry, organized psychiatry, governmental agencies and the corporate health sector need to come together to develop initiatives and programs to provide both the required psychiatric education as per MCI guidelines, and education in/exposure to cutting-edge developments in neurosciences and psychological therapies.

(a) Development of a network of psychiatric educators from within India and abroad with one to two-week, state-wide intensive training courses held at a central location, (b) development of faculty networks within India with aid and assistance from organizations and foundations for medical education, such as the FAIMER Institute, the Centre for Medical Education and Technology at the All India Institute of Medical Sciences, etc[54,55] (c) use of tele-psychiatry education methods – many projects using tele-methods are currently ongoing in India and can be adapted for medical education, for example the schizophrenia research foundation (SCARF),[56] has a telemedicine program for consumer advocacy and education on schizophrenia, Kakatiya University in Warrangal, Andhra Pradesh has developed many courses for tele-education at its school of distance learning and continuing education,[57] (d) online courses using e-technology such as the Blackboard.[58] As an example, it is noted that Cambridge College in England is starting an e-course in Business through a satellite college in Mumbai,[59] (e) funding student travel to psychiatry meetings, (f) two-week studies at colleges of excellence within India, (g) private courses similar to the national entrance examination preparation courses for medical colleges, Indian Institutes of Technology etc, (h) creation of student psychiatry societies and observing a Psychiatry Day, in medical colleges, (i) one-week psychiatry institutes in the summer holidays for interested students, (j) development of language and culture-sensitive psychiatric curriculum in each state, etc are some of the viable methodologies that deserve further consideration. The summit delegates also focused on (k) models of excellence in teaching as one such method. The Kochi neuropsychiatry workshop conducted by joint faculty from the US and India in 2007 was mentioned as an example.[60] It is plausible and necessary to develop many faculty teams to meet the needs of the numerous second and third tier colleges and wide geographic distribution of colleges. The following goals were prepared:

### Objective II - Goals

Formation of a medical education work-group from among the summit delegates and additional invited members with interest and track record in medical education from interested organizations to liaison with the UG Education taskforce of IPS to examine in depth the challenges faced by previous advisory groups and develop specific methods of achieving this objective at the levels of the individual institution, state/university and national. Subgroups from this larger taskforce to identify potential funding sources and opportunities and propose/implement specific projects utilizing the techniques mentioned above. Similar to the proposed public and leadership education consortium, this UG medical education taskforce should strive to develop a consortium of advocates for policy and curricular changes and teaching methods. On a more pressing note, the GOI should be lobbied and MCI persuaded to include psychiatry as a separate subject for examination in UG education.

An early goal for this taskforce is encouraging partnerships between available and willing psychiatric educators from the US (and other countries) and India, and creating sister-institution relationships for broadly strengthening the elements of UG curriculum in psychiatry. It is recommended that focused efforts be dedicated using the models and tools listed above, in assisting second and third level medical colleges/institutions to improve UG education in Psychiatry.

### Objective III - Postgraduate and continuing medical education

The first HCS had concluded that much variability existed in the postgraduate curricula in psychiatry within non-medical disciplines, and was virtually nonexistent in non-psychiatric medical disciplines, such as Medicine, Obstetrics and Gynecology etc. In this summit, education and training of three graduate trainee groups were briefly reviewed, namely psychiatrists, non-medical MH disciplines (psychology, psychiatric social work), and non-psychiatric physicians. It was reiterated that there is hardly any teaching of psychiatry during residency training in non-psychiatric disciplines, although it is widely known that co-morbid psychiatric disorders and psychosomatic conditions are fairly prevalent, that psychiatric disorders often masquerade as somatic disorders, and somatic disorders may be misdiagnosed as psychiatric conditions.[61–64]

Opportunities for continuing medical education (CME) and professional development (CDP) of physicians are limited and not formalized. Although a committee of MCI exists for CME since 1985, the total number of CME activities are limited in relation to the need, and there is no formal requirement for CME, especially for medical license renewal.[65] As an aside, a certain amount of CME activities for each specialty appear to be organized by Pharma. However, recently, the role and motivations of Pharma in this regard are being questioned, in India and abroad.[66] Professionals need to be sensitive to the perceptions of the public and regulatory authorities on this issue. Therefore, it is not clear how much support and in what form could be sought from these entities. Yet they remain a potential funding source. In the US, several ideas have been proposed to maintain Pharma funding while reducing conflicts of interest for faculty and practitioners, and retaining control of the scientific content by the profession. For example, a centralized, non-profit education agency into which Pharma may contribute. Through such ideas, it may be possible to bring about an alliance between Pharma, Academia, Educational authorities and practitioners, to the mutual benefit of all.

Psychiatry postgraduate training programs do an excellent job in training psychiatric specialists, given the limited resources made available to them. The biggest challenge is simply the limited number of programs offering psychiatric specialization, the limited number of physicians who choose to go into psychiatry for a career, and consequently the very small number of psychiatrists in the country, estimated to be around 3000. As a corollary to this, the number of psychiatrists available to train future psychiatrists is even smaller, and is concentrated in the top 20-30 institutions of the country. In related fields such as Clinical Psychology, and Mental Health Social Work, the situation is even worse with fewer programs across the country. Education Training in co-morbid psychiatric disorders, especially depression and anxiety disorders for non-psychiatrist generalists and specialists is negligible or absent. Thus, the needs in these groups are simply enormous. There is a need for a core curriculum in psychiatry for implementation during postgraduate training of various medical specialists, especially disciplines such as internal medicine, obstetrics and gynecology, pediatrics, and neurology, and for clinical psychologists, and MH social workers.

As the interface of Psychiatry and Medicine grows, it is also important that joint CME programs with a psychiatrist and a non-psychiatrist physician be encouraged. For non-psychiatry medical generalists and specialists in CME programs, it was suggested that joint CME programs with a psychiatrist and a non-psychiatrist physician be encouraged. Faculty from the US (and other countries) could play a valuable role in “training the trainers” in India to develop a cadre of CME-CPD trainers. Models being pursued by the Royal College in UK and the American Board of Psychiatry and Neurology in the US need to be imported and indigenized for practitioners in India.

### Objective III - Goals

Development of uniform and strong curricula for each group of trainees, including postgraduate training of psychiatrists, postgraduate training of various medical specialties, especially General Medicine, and its specialties, Neurology, Pediatrics, and Obstetrics and Gynecology, postgraduate training of non-medical MH disciplines such as clinical psychology and social work. In the pursuit of this goal, the summit leadership should join forces with IPS, and academic departments within India, and liaison with entities abroad such as the Accreditation Committee for Graduate Medical Education in the US, and the Royal College of Psychiatry in the UK. The group should work with the Postgraduate Education Committee of MCI and at the state level with medical universities, and individual institutions. The group should actively encourage and support faculty visits, joint appointments, travel fellowships, resident exchanges, etc to create a greater awareness of this need amongst all the stakeholders and decision-makers. Further, the above efforts will create the needed infrastructure to implement agreed-upon curricular changes including development of uniform curricula, and for direct teaching efforts. Mechanisms similar to the ones noted under UG education may be utilized.

Further, it is recommended that we work with MCI to develop criteria for continued licensing including a specific number of credit hours for CME/CPD, as is the case in all developed countries.[67] For non-psychiatry specialties, we should work with MCI and the respective professional organizations to advocate a minimum number of psychiatry CME/CPD hours, annually.

### Objective IV - Reduce complications of depression (suicide, alcohol, etc.)

#### Suicide

The background information on suicide in India and Asian countries that was presented in the first summit was reviewed, and updated. Suicide is a complication of depression. Suicide rates in India have been reported in the range of 98–118 per 100,000. These are likely underestimates.[3–6,68] It was noted that despite previous attempts to repeal the article of the penal code making suicide attempt a crime, this law remains on the books. It is a significant impediment in urgent clinical assessments and providing timely assistance to victims of attempted suicide, and in data gathering. Most recently, the Law Commission of India in its report on Humanization and Decriminalization of Attempted Suicide has strongly recommended the repeal of this law.[69] The MH panel strongly supports this recommendation and asks the GOI to expeditiously initiate the necessary steps. The commonest methods of suicide are hanging and use of organo-phosphorous poisons.[6] When correctly identified as a suicide attempt, brief interventions starting within the emergency room and follow-up can significantly reduce future suicide.[70] Many vulnerable populations have been identified such as women exposed to violence, poverty and having a mental illness, and new groups are emerging such as persons working in call centers, and farmers burdened with heavy debt.[71–75]

Recommended prevention strategies advocate both individual specific interventions by professionals and trained lay persons (www.befrienders.org/helplines/helplines)[70,76] and population-wide interventions.[77] Whereas there are many studies on suicides in India, there is no coordinated strategy, national policy or state-wide effort in the public or the NGO sector, as noted in an editorial by Manoranjitham, Abraham and Jacob.[78] This editorial also suggests a series of specific steps towards developing and implementing such a policy.

#### Alcoholism

This objective had been added to the agenda of the second summit. The group felt that it is important to emphasize the relevance of assessment and treatment of alcohol use problems to all medical specialty practice, both as a specific clinical issue and for sensitizing the medical community to this public health problem. There is no dearth of studies on the relation between alcoholism and specific medical disorders. As many as 60 different medical disorders have been more or less specifically associated with alcohol abuse and the public health and social impact of alcohol use is massive.[79,80] Specifically, our colleagues need to be educated that alcoholism is often a link between mental health disorders and other medical conditions.[81,82] There is good data to suggest that brief interventions by providers can have a substantial positive effect in addressing alcoholism and extended rehabilitation is not always necessary.[83] Manuals are available for the benefit of medical officers dealing as first responders to alcohol-related medical-psychiatric conditions. The panel also called for increased research on (i) the relation between alcoholism and suicide and (ii) on depression and associated alcoholism in the younger population, (iii) alcohol dependence and risk behaviors such as road accidents and other mishaps, and (iv) alcohol abuse and depression as precipitating and perpetuating factors in domestic violence.

### Objective IV - Goals

- Advocate for the expeditious repeal of Section 309 of the Indian Penal Code, emphasizing humanitarian and data collection reasons amongst others.
- Collaborate in the development of suicide assessment and intervention training programs adaptable for the community, general practice, clinic and hospital levels.
- Initiate collaborations on pilot studies to identifying vulnerable groups/areas in India such call centre and IT sector employees, farmers, and rural women in poverty etc and conduct assessment of suicide risk. Develop studies on vulnerable group-specific individual and group intervention models.
- Advocate for inclusion of depression, psychosis, alcoholism, and suicide in the national data registry (IDSP).
- Develop, update and/or customize educational material and programs for the medical fraternity on the link between alcoholism and medical disorders

### Objective V - Integrating MH care and primary health care

It was noted that depression is about 1.5 times more prevalent in medically co-morbid patients than those without such co-morbidity.[7,84] The relative risk of cardiac mortality in the presence of depression increases threefold in patients with existing cardiac conditions and fourfold in those without previous cardiac morbidity.[7] Integrating medical and psychiatric care has long been advocated, and although there has been unanimity that such integrated service is desirable, there have been many challenges to actual implementation and practice[85–89] in all countries. For example, primary care physicians may be concerned about losing control of their practice or being stigmatized by seeing too many psychiatry patients. In one of the earliest training attempts conducted in India, only 10% of the trained general practitioners were thought to likely use the training in their practices.[89] In public health systems, efforts at integration could lead to unanticipated effects such as withdrawal of primary care providers from the system.[87] Since the late ‘70s, there have been attempts in India in integrating psychiatric care with primary health care in rural areas through the training of multipurpose health workers as in the Raipur Rani Experience,[90] and in training general practitioners.[88,91] There are many promising models of training medical students and non-psychiatric residents in integrated care to lay the ground for successful integrated care in their later practices.[92–96] Effective integration is possible and requires careful and pragmatic planning. There is an ongoing debate about the relative utility of collaborative disease-centered models versus direct-to-patient person-centered models.[96–102]

The summit members focused on the training aspects of family physicians and primary care providers (general practitioners), in mental health and specifically depression. Reference was made to the availability of specific training modules for this purpose, for example the IMPACT project developed largely by the Washington University, Department of Psychiatry to train primary care physicians in the identification, assessment and treatment of depression in a primary care setting. This program has both in-person and online versions and is available free to interested organizations, practices and institutions.[103] Such training may be achieved by several methods. Firstly as had been discussed above, under PG training of other specialties.[93,94,104] Secondly, through the use of ongoing CME. Several specialized and brief scales are available for integrated care, for example the PRIME-MD and the PC-SAD[105,106] and the utility of the PRIME-MD in the Indian setting has been demonstrated.[107] Further, there is some evidence to suggest even shorter screening with two or four questions may provide sufficient sensitivity in detecting depression and anxiety in the general medical setting.[108,109] The Institute of Human Behavior and Allied Sciences (IHBAS), Delhi, has also been coordinating a multi-site project on ‘Urban Mental Health Problems and their Service Needs’. As a part of this project, IHBAS has been developing modules including a Depression Intervention Module and conducting training programs for general practitioners on various mental disorders.

The use of electronic media and telemedicine for quick and long-distance dissemination of information was once again emphasized to reach remote practitioners. Further, it is important to utilize process indicators and objective outcome measures to update and modify training methods based on actual needs and utilization by practitioners.

### Objective V - Goals

- Development of or suitable modification and updating of existing ‘psychiatry for primary care’ training and practice modules for use by general practitioners (GPs) in India.
- Development of or suitable modification and updating of existing training and practice modules for use by GPs in India, on the topics of alcoholism and suicide.
- Collaboration through direct participation in ongoing projects, and initiate new projects, especially through academic departments, NGOs, and IPS, in urban and more importantly semi-urban and rural areas.

## CONCLUSIONS

Healthcare summits such as that reported in here and recommendations arising from the same can serve as a springboard for concerted action by all the stakeholders. It is hoped that this summit and report will serve such a purpose. The four broad areas selected by the summit for deliberation are only a small piece of the monumental challenges that face consumers, providers and advocates of psychiatric care in India. While some progress has been made and some is planned or is in progress, much more is needed. It is believed that harnessing all available resources, especially experience and expertise gathered abroad by psychiatrists of Indian origin may be put to good use in bringing the best to our patients.

## PARTICIPANT LIST - INDO-US HEALTH CARE SUMMIT 2009 MENTAL HEALTH DELEGATES

India – Nimesh Desai, Chair, MAM Khan, co-chair, Rajesh Nagpal, Smitha Deshpande, S.C. Mullick, Samir Parikh, Rajesh Sagar, Rashmi Chaudhry, Jugal Kishore, Sanjay Gupta, Nikhil Raheja, Y Matcheshwalla, Deepak Kumar, Rajesh Kumar, Saurabh Malhotra, and Pallavi Dham.

USA – Anand K. Pandurangi, Chair, Shiv Hatti, Co-chair, Surinder Nand, Meera Narasimhan, Arif Khan, and Satish Shrikhande.
